# Worse Than the “Maine Law”

**Published:** 1855-11

**Authors:** 


Worse than the "Maine Law."—The town of Clonmel, in Ireland, has lately enacted an edict worthy to figure in the code of Lycurgus. Every person detected in a state of intoxication will be led or carried, according to the gravity of the case, to the municipal prison; after which the nearest apothecary will be required to empty his stomach by a pump *ad hoc.* The operator will receive a fee of seven shillings and six pence. If this plan does not cure the intemperate, it will at least benefit the apothecaries.—*Ib.*

A Case of deep Wound of the Parorid Region, in which a Ligature was simultaneously applied to the Common and Internal Carotid Arteries. By Gurdon Buck, M. D., Surgeon to the New York Hospital.—Wm. McGraw, a laboring man, aged thirty years, and a native of Ireland, was admitted, July 5, 1848, into the Hospital with a deep wound in the right parotid region, that happened the day before from the explosion of a glass bottle containing gunpowder. The patient was holding the bottle at the time of the accident. The hæmorrhage, which was profuse, was arrested with much difficulty by sewing up tightly the external wound, after first extracting a fragment of glass one inch in length. The upper part of the neck and cheek, surrounding the wound, were a good deal swollen and tense. The wound itself was scarcely half an inch in length, and was situated half an inch anterior to the lobe of the ear. After enlarging its orifice, the finger passed directly inwards behind the ramus of the jaw, a depth of nearly two inches, and could be moved about with some freedom at the bottom of the wound. No hæmorrhage followed the exploration. The patient's mouth was drawn towards the left side, and much distorted. Deglutition was difficult, and his voice, hitherto distinct, was thick and not easily understood. Patient was directed to keep in bed, and to have cold water dressings applied to the part.

*July* 8th. On careful examination, the swelling, which had increased, presented a distinct pulsation at its central and most prominent part, showing conclusively the development of a false aneurism.

9th. At four o'clock, A. M., a very profuse hæmorrhage occurred, which could not be controlled by pressure over the wound, but was finally arrested by very firm compression applied to the common carotid. After being kept up by the Resident Surgeon and his assistants for nearly two hours, compression with sponge in the wound was substituted, and applied in the following manner. Three pieces of compressed sponge were successively passed down into the capity of the wound, and a fourth placed over the orifice. Two graduated compresses were applied over the sponges, and the whole maintained in place by the finger of an assistant. The patient was thus relieved of the painful pressure over the neck, and time was allowed to convene the Surgeous for an operation.

*Operation* at 9, A. M. For reasons that will be stated hereafter, it was decided to apply a ligature to the internal as well as the common carotid artery. The procedure was as follows:—

An incision three inches in length was made from a point a little above the ramus of the os hyoides downwards over the edge of the sterno-mastoid muscle. This muscle being laid bare, its anterior edge was raised from its sheath and drawn outwards. A transverse venous branch that crossed the carotid at the point where it was to be tied. was secured by two fine ligatures and cut between them. The sheath of the common carotid was next opened; and a ligature passed beneath the artery, but not tied. The dissection was now carefully prosecuted upwards to the bifurcation, and the internal carotid exposed to the extent of three-fourths of an inch from its origin. In the first attempt to pass the armed needle under the artery, the patient became suddenly convulsed, his breathing stertorous, and his pulse feeble, evidently from disturbance of the pneumo-gastric nerve. Further proceedings were suspended to allow these alarming symptoms to subside, which required five or six minutes. A second attempt to pass the ligature was then successful without any accident. Before tying the knot a careful examination was made to ascertain that nothing was included in the ligature but the artery. The effect of cutting off the current of blood from the brain was also tested by tightening the thread over the finger placed in the noose with the artery. The ligature was then tied in the usual manner, and that around the common carotid was also secured an inch below its bifurcation and above the omohyoid muscle. The external wound was closed with sutures, and between them strips of muslin wet with collodion were applied. Ice-water dressings were directed.

*July* 11th. Patient is progressing favorably, his pulse is one hundred, full and strong. The swelling of the parotid region has diminished. Pus mixed with blood is freely secreted from the original wound.

14th. The swelling has still further subsided, and the suppuration from the original wound very much diminished.

20th. The ligature has come away from the internal carotid. No pulsation is perceptible either in the facial or temporal arteries of the right side.

21st. The ligature came away from the common carotid.

*Aug.* 5th. Patient has complained for the last four or five days of severe pain over the right side of the head and face, anterior to the ear, and has had frequent slight hemorrhage from both nostrils. The wounds have nearly healed. No excitement of pulse. Ordered cups and scarifications to the temples.

*Aug.* 12th. Since the last report blisters have been applied behind both ears, and with decided relief of the pain in the head. The epistaxis has recurred but once, and only in a slight degree. His bowels are constipated.

14th. The pain in the head has returned this morning with great severity. Constipation still persists in spite of active purgatives. Ordered a blister to be established on the forehead with aq. ammon. fort.; and croton oil, gutt. ij. to be taken, and followed by enema if necessary.

15th. The head is much relieved. The bowels have been freely evacuated. The blister to be continued behind the ear.

26th. The patient's general condition is improving; his bowels, however, still continue obstinately constipated, and require the most active purgatives to move them. The wounds have not yet entirely healed; that in the parotid region is converted into a fistula, from which saliva flows freely, especially during the act of mastication. The mouth, when opened, is still drawn to the left side; the right half of the tongue has become atrophied and flabby, presenting longitudinal wrinkles on its surface. Its appearance strangely contrasts with that of the left half, which retains its natural, plump and healthy condition.

*Sept.* 21st. Everything continued to progress favorably till this morning, when patient became feverish, and sick at his stomach. During an effort to vomit he was suddenly startled by a gush of arterial blood from the wound in the neck, amounting to about two ounces. Before assistance could reach him the hemorrhage ceased spontaneously.

*Oct.* 18th. No return of hemorrhage. The salivary fistula noticed above has closed under the influence of pressure applied with the end of the finger to the orifice of the fistula during meals. Patient was discharged cured this day.

Several months afterwards, when he presented himself for examination, the right half of the tongue was still found atrophied and shriveled, though the function of taste remained unimpaired. The mouth was drawn to the left side, distorting the face. His articulation continued thick and indistinct.

*Remarks.*—The situation and depth of the wound in the above case, as well as the profuseness of the hemorrhage and the extreme difficulty of controlling it by pressure, plainly indicated that a large artery had been divided. The external carotid artery and certain of its branches, as well as the internal carotid, lay close cogether in the region occupied by the track of the wound, and it was impossible to say which of them might be involved.

In the choice of means for arresting permanently the hemorrhage, the following considerations were carefully weighed:

1st. In reference to securing the divided vessels in the wound. This undoubtedly would have been the most reliable method, and is the one prescribed by the rules of surgical practice. It is not, however, always admissible. Obstacles may exist, depending on the inaccessible location of the wounded vessel, or other equally insurmountable difficulties. In this case the wounded vessels lay deep in a narrow space bounded anteriorly by the ramus of the lower jaw, and posteriorly by the mastoid process and the sterno-mastoid muscle, which presented unyielding barriers that forbade any distention of the edges of the wound, even had it been admissible to enlarge it by incision. The exposure of other larger vessels to be wounded in attempting this operation, and the extreme difficulty of controlling the hemorrhage by compression of the common carotid during the operation; these were all weighty reasons that deterred from resorting to this method.

2d. Ligature of the common carotid. This is the resource heretofore most generally relied upon in wounds of the neck, when tying the wounded vessels has been inadmissible. Though it has succeeded in many cases, yet it has not unfrequently proved ineffectual, and that oftener, no doubt, than the records of surgery disclose. The free communication between the internal carotids of either side through the circle of Willis, permits a return current to be promptly established through the internal into the external carotid and its branches of the same side, after the ligature of the common trunk. This was illustrated in the writer's own experience a few years ago in a case of self-inflicted wound of the throat, exposing the left thyroid cartilage. A profuse secondary hemorrhage occurred at the expiration of five or six days, for which the common carotid artery was tied. One hour had scarcely elapsed before the hemorrhage recurred almost as profusely as at first. Compression kept up by means of a succession of assistants was relied on for three or four days longer, when a third repetition of the hemorrhage rendered it necessary to resort to other means. The original wound was now enlarged and explored, and after much difficulty the bleeding vessel was secured. It was found that a small false aneurism had developed itself at the bottom of the wound around the divided vessel. Another consideration that dissuaded from the operation of tying the common carotid artery alone in this case, was the fear entertained that the internal carotid might itself be wounded.

The novel operation which was resorted to in this instance and which was so successful, was thought to be adapted to any contingency that might exist. By cutting off simultaneously the direct current through the common carotid and the return current through the internal carotid, hemorrhage from a wounded branch of the external carotid would be effectually controlled; and in the event of a wound of the internal carotid, the circulation would be rendered stagnant in that vessel by the ligature applied to it near its origin, and the process for its permanent closure would be secured by the simultaneous ligature of the common carotid.

Other applications of this operation suggest themselves, and claim the serious consideration of practical surgeons; for instance, where destructive ulceration of the fauces invading the internal carotid or other arteries had given rise to alarming hemorrhage, it would be the surest reliance.

In the *London and Edinb. Monthly Journal of Med. Science*, vol. for 1842, p. 961, Mr. Syme has reported a remarkable case of aneurism of the internal carotid, which was recognized as such by a pulsating tumor in the region of the tonsil, and in which he had tied the common carotid artery. The patient died suddenly and unexpectedly in thirty hours after the operation. The post-mortem examination corroborated the diagnosis, but did not explain the cause of death. Mr. S. remarks in reference to this case, that "the pulsation of the tumor continued after the operation, but was much less forcible." . . . . "The contents of the sac were coagulated, except at a narrow channel corresponding with the current through the artery, which, it may hence be inferred, had not been completely arrested. Indeed this was not and could not reasonably be expected when the free retrograde passage afforded by the anastomosing communications of the external carotid was taken into account."

Mr. Syme's remarks would apply with much greater force to the anastomosing communications between the internal carotids through the circle of Willis, by means of which the return current would be much more promptly established than through the anastomoses of the external carotids. To such a case as Mr. Syme's our operation, it is thought, would have been pceuliarly applicable.

The atrophy of the right half of the tongue, following the operation in the case just narrated, is an interesting point, and one which might perhaps suggest the expedient of tying the lingual arteries as a remedy for hypertrophy of the tongue.—*New York Medical Times.*

